# Rectangular Transition Metal-rTCNQ Organic Frameworks Enabling Polysulfide Anchoring and Fast Electrocatalytic Activity in Li-Sulfur Batteries: A Density Functional Theory Perspective

**DOI:** 10.3390/molecules28052389

**Published:** 2023-03-05

**Authors:** Jie-Zhen Xia, Lu-Chao Zhao, Man-Hua Liao, Qi Wu

**Affiliations:** 1 School of Science, Tibet University, Lhasa 850000, China; 2 Institute of Oxygen Supply, Center of Tibetan Studies (Everest Research Institute), Tibet University, Lhasa 850000, China; 3 Key Laboratory of Cosmic Rays, Tibet University, Ministry of Education, Lhasa 850000, China

**Keywords:** metal-organic frameworks (MOFs), Li-S batteries, polysulfides, TM-rTCNQ

## Abstract

Two-dimensional metal-organic frameworks (MOFs) have shown great development po-tential in the field of lithium-sulfur (Li-S) batteries. In this theoretical research work, we propose a novel 3*d* transition metals (TM)-embedded rectangular tetracyanoquinodimethane (TM-rTCNQ) as a potential high-performance sulfur host. The calculated results show that all TM-rTCNQ structures have excellent structural stability and metallic properties. Through exploring different adsorption patterns, we discovered that TM-rTCNQ (TM = V, Cr, Mn, Fe and Co) monolayers possess moderate adsorption strength for all polysulfide species, which is mainly due to the existence of the TM-N_4_ active center in these frame systems. Especially for the non-synthesized V-rCTNQ, the theoretical calculation fully predicts that the material has the most suitable adsorption strength for polysul-fides, excellent charging-discharging reaction and Li-ion diffusion performance. Additionally, Mn-rTCNQ, which has been synthesized experimentally, is also suitable for further experimental con-firmation. These findings not only provide novel MOFs for promoting the commercialization of Li-S batteries, but also provide unique insights for fully understanding their catalytic reaction mecha-nism.

## 1. Introduction

With the ever-increasing development of society and the demand for energy storage systems, lithium-sulfur (Li-S) batteries (LiSBs) have been extensively investigated and considered as gifted candidates to replace Li-ion batteries [1], due to their extremely high energy density (~2600 W h kg^-1^) and theoretical specific capacity (~1675 mA h g^-1^) [2,3,4,5,6,7]. Another reason for the popularity of LiSBs is that sulfur hosts possess many advantageous attributes, such as low cost, high earth-abundance, non-toxicity and environmental benig-nity [6,7,8,9]. Despite these promising features, some persistent issues have been greatly re-stricting the commercial application of LiSBs. These problems mainly boil down to the following aspects [2,10,11,12,13,14]: (1) low electrical conductivity of elemental sulfur and the short-chain Li_2_S_/_Li_2_S_2_ species, (2) the shuttle effect of the dissolved long-chain lithium pol-ysulfides (LiPSs), and (3) the sluggish charge-discharge reaction kinetics of whole electrochemical reaction process.

In this context, much attention has been focusing on exploiting novel cathode mate-rials [15], which can facilitate the LiPSs conversion reactions and thus increase the sulfur utilization and cycle lifetime of LiSBs. For this purpose, a successful approach requires the utilization of polar materials including doped graphene [16], metal nanoparticles [17], metal-based compounds (e.g., borides [18,19], carbides [20], nitrides [7,21,22], oxides [23,24,25], phosphides [26,27,28,29], sulfides [30,31,32]), defective borophene [33] and phosphorene [34], MXenes [2,35,36], metal organic frameworks (MOFs) [14,37,38,39,40], etc, as potential LiSBs host materials. Among these candidates, two dimensional (2D) MOFs displaying the charming features of large surface area [41,42] and tunable aperture sizes, have been highly concerned as one hopeful choice for sulfur hosts. However, research on the catalytic performance and mechanisms of these materials is still in its infant stage. Recently, various of 2D metal-7,7,8,8-tetracyanoquinodimethane (TCNQ) molecule coordination networks and TM-based 2D TCNQ monolayers have been synthesized [43,44,45,46,47,48,49,50]. Owing to the unique tetragonal (mainly rectangular and square) 2D lattice of atomic thickness, TCNQ has become an important building block for constructing novel func-tional nanomaterials with diverse properties (magnetic, optical, mechanical, electronic, etc). Furthermore, many theoretical studies have also shown that the TM-rCTNQ framework has great application potential in the NRR [51], ORR/OER [52], CO_2_RR [53] and other electrocatalytic fields [54], but it has never been reported in the direction of LiSBs. On the basis of these exciting results, we speculate that TM-TCNQ can also be served as a potential anode material for improving the LiSBs performance. 

Herein, we systematically explored the application of 2D TM-based rectangular TCNQ monolayers (referred to as TM-rTCNQ) in LiSBs based on first-principles calcula-tions. In addition to the structure that has been synthesized experimentally (Mn-rTCNQ [49], Ni-rTCNQ [47]), we also studied the performance of other 3*d* family TM-based rTCNQ (TM = Sc, Ti, V, Cr, Fe, Co and Cu) as highly efficient electrocatalysts for LiSBs. It was found that TM-rTCNQ (TM = V, Cr, Mn, Fe and Co) monolayers not only exhibit excellent metallic nature, but also have moderate binding strengths to polysulfides. Re-markably, the low energy barriers of the Li_2_S decomposition are reduced to 0.88–1.41 eV, while the Li-ion diffusion barriers are in the range of 0.23–0.53 eV on TM-rTCNQ (TM = V, Cr, Mn, Fe and Co) monolayers, ensuring the rapid catalytic redox of LiPSs during the charge-discharge process. In a word, V-rTCNQ, as the most promising MOFs of all 3*d* family TM-rTCNQ structures, is very worthy to be prepared and have its performance verified, and the synthesized Mn-rTCNQ is also ready to be further confirmed experimen-tally. These findings offer a deep insight into the catalytic effects of novel 2D MOFs, as well as a promising strategy and general guidance for designing LiSBs.

## 2. Results and Discussion

### 2.1. Structure, Stability and Electronic Properties of TM-rTCNQ and S_8_/LiPSs

**Figure 1:**
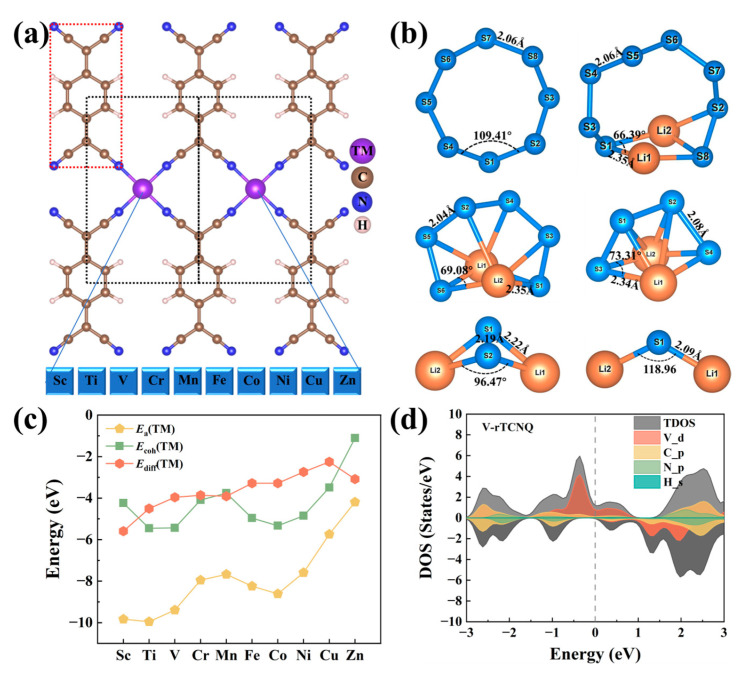
(**a**) Schematic diagram of TM-rTCNQ (TM = Sc, Ti, V, Cr, Mn, Fe, Co, Ni, Cu and Zn) geometric structures (the red and black rectangle boxes represent the TCNQ molecule and unit cell of TM-rTCNQ, respectively). (**b**) Fully optimized S_8_/LiPSs molecules and some structural parame-ters are marked at corresponding positions. (**c**) The calculational results of adsorption energies (*Ea*), cohesive energies ($E_{coh}$ ) and $E_{diff}$ ($E_{a}-E_{coh}$ ) of TM-rTCNQ. (**d**) The DOS diagram of V-rTCNQ unit cell, including total DOS (TDOS), projected DOS of *d* orbital of V (V_d), *p* orbital of C and N (C_p and N_p), as well as *s* orbital of H (H_s). Fermi-level is at the position of the gray dotted line.

The schematic diagram of the 2 × 1 × 1 supercell of all 3*d* family TM-rTCNQ (TM = Sc, Ti, V, Cr, Mn, Fe, Co, Ni, Cu and Zn) structures is shown in Figure 1a, and black/red rectangular box areas represent the TM-rTCNQ unit cell and TCNQ molecule, respect-tively. Among them, each 3*d* TM atom is combined with four N atoms in TCNQ clusters. The detailed structural information is listed in Table S1. As observed in Figure S1, all at-oms lie in the same plane of TM-rTCNQ (TM = Sc, Ti, V, Cr, Mn, Fe, Co and Zn) mon-olayers. Some structural distortion of TM-N_4_ center is observed for Ni-rTCNQ and Cu-rTCNQ, which is also consistent with related research works [51]. In addition, all the S_8_/Li_2_S*_x_* (*x* = 1, 2, 4, 6, 8) species are fully optimized (Figure 1b) and are highly consistent with the previously reported theoretical calculations [16,55,56].

Additionally, the stability of these catalysts is evaluated by the energy difference ($E_{diff}$) between adsorption energies ($E_{a}$) and the average cohesive energies ($E_{coh}$). Gener-ally, systems with $E_{diff}$ = $E_{a}-E_{coh}$ < 0 represent that TM atoms can be atomically dis-persed on the TM-rTCNQ monolayers through the coordination effect. As shown in Fig-ure 1c, the *E*_a_(TM) of TM-rTCNQ is within the range of −4.19–−9.95 eV, which indicates the strong interaction between TM atoms and the N_4_ active center. The negative values of $E_{diff}$ reflect that all TM-rTCNQ configurations have sufficient structural stability to meet the possibility of experimental preparation. In addition, excellent electrical conductivity of the sulfur host materials is an important factor for practical Li-S battery applications. We focused on the conductivity of TM-rTCNQ and calculated the density of states (DOS) of all TM-rTCNQ unit cells, as shown in Figure 1d and Figure S2. The TM-rTCNQ (TM = Sc, Ti, V, Cr, Mn, Fe, Co, Ni and Cu) structures, except for Zn-rTCNQ, all exhibit metallic prop-erties and are expected to improve the electrochemical performance of Li–S cells.

### 2.2. Adsorption Performance of TM-rTCNQ for S_8_/LiPSs

All well known, 1,3-dioxolane (DOL) and 1,2-dimethoxyethane (DME) electrolyte molecules can yield a competitive trapping to soluble Li_2_S_4_, Li_2_S_6_ and Li_2_S_8_. Therefore, the adsorption energies of Li_2_S_4_ (Li_2_S_6_, Li_2_S_8_) anchored on TM-rTCNQ substrates were first calculated. As displayed in the Figure S3, the energy range is from −0.88–−0.77 eV and mainly depends on Li-O bond interactions, which is highly in accordance with previous works [3]. An optimal host material should trap the soluble LiPSs more strongly than the DOL/DME solvent molecules to restrain the shuttle effect.

**Figure 2:**
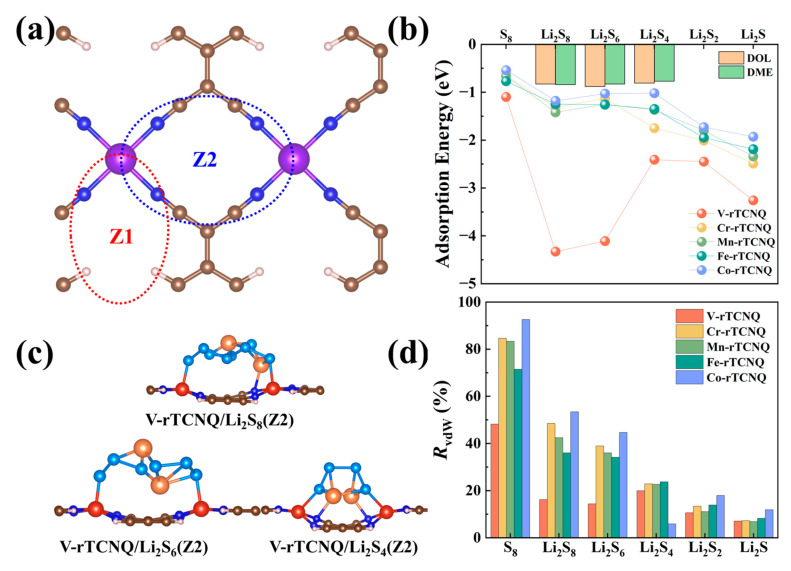
(**a**) Schematic diagram of Z1 and Z2 adsorption patterns. (**b**) The adsorption energies of TM-rTCNQ (V, Cr, Mn, Fe and Co) structures for S_8_/LiPSs. (**c**) The top and side views of V-rTCNQ adsorbing Li_2_S_4_, Li_2_S_6_ and Li_2_S_8_ in Z2 pattern. (**d**) The ratio of van der Waals (*R*_vdW_) interaction of TM-rTCNQ (V, Cr, Mn, Fe and Co) adsorbing S_8_/LiPSs clusters.

Herein, the adsorption energies of all TM-rTCNQ for polysulfides were calculated and shown in Figure 2b, Figures S4 and S5. We considered two adsorption patterns (Z1 and Z2). As shown in Figure 2a, Z1 mode involves trapping the LiPSs in the center of TM-N_4_ coor-dination network, and Z2 mode refers to adsorbing the LiPSs with two TM atoms of the TM-rTCNQ supercell. It can be seen that Sc-rTCNQ and Ti-rTCNQ exhibit significant binding and structural distortion for S_8_ and soluble LiPSs through Z2 mode due to the strong interaction between two TMs and S atoms. Moreover, our results also show obvi-ous structural changes for the Ni-rTCNQ/Li_2_S(Z1) and Cu-rTCNQ/Li_2_S(Z1) adsorption systems. Therefore, TM-rTCNQ (TM = Sc, Ti, Ni and Cu) monolayers are ruled out since the structural integrity of the host is essential to the subsequent reactions. For the remain-ing TM-rTCNQ (TM = V, Cr, Mn, Fe and Co) structures, the adsorption energies of the most stable configurations for S_8_/LiPSs on TM-rTCNQ are summarized in the Figure 2b. Obviously, these structures can effectively inhibit the shuttle effect problem and V- rTCNQ possesses the most outstanding adsorption performance shown in Figure 2c.

Finally, the ratio of van der Waals (*R*_vdW_) effect is shown in Figure 2d to further ana-lyze the physical or chemical interactions between TM-rTCNQ and polysulfides. Accord-ing to Figure 2d, one can see that the effect of chemical binding is relatively increased along with the progression of the lithiation process. For example, the weight of vdW in-teraction contributing to the adsorption energy is 83.33% for the Mn-rTCNQ/S_8_ system, whereas for Mn-rTCNQ/Li_2_S, this ratio decreases to 6.84%. In addition, the vdW chemical interactions play a dominant role in the early transition metal decorated V-rTCNQ, with *R*_vdW_ in the range of 7.06–48.18%, leading to an impressive anchoring effect on S_8_/LiPSs. Among them, the *R*_vdW_ of V-rTCNQ/Li_2_S_6_ and V-rTCNQ/Li_2_S_8_ adsorption systems is lower than that of V-rTCNQ/Li_2_S_4_, which is significantly related to the extremely strong adsorp-tion energy of V-rTCNQ for Li_2_S_6_ and Li_2_S_8_ clusters.

### 2.3. Electronic Structure Analysis 

**Figure 3:**
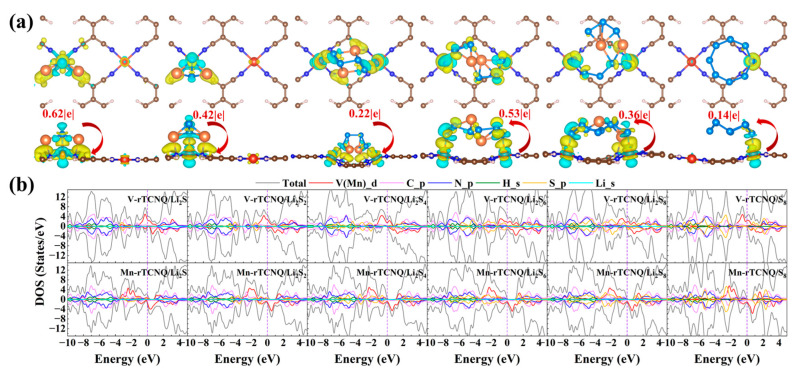
(**a**) The charge density differences and the charge transfer amount of the adsorption sys-tems of V-rTCNQ for S_8_/LiPSs. (**b**) The total and projected DOS of V-rTCNQ and Mn-rTCNQ after anchoring S_8_/LiPSs clusters. Fermi-level in the violet dotted line is set to be zero.

In order to further reveal the interactions of TM-rTCNQ configurations anchoring S_8_/LiPSs, the charge density differences (Δ*ρ*) and DOS were calculated. The Δ*ρ* and charge transfer amount of all adsorption systems of TM-rTCNQ (TM = V, Cr, Mn, Fe and Co) for S_8_/LiPSs are displayed in the Figure 3a and Figure S6. One can see that the Li atoms of LiPSs form bonds with the N atoms on the V-rTCNQ surface, whereas the V atoms mainly bond with the adjacent S atoms of S_8_/LiPSs, as shown in Figure 3a. Moreover, Li_2_S, Li_2_S_2_ and Li_2_S_4_ donate electrons to the V-rTCNQ host, whereas the high order Li_2_S_6_, Li_2_S_8_ and S_8_ act as acceptors with the increase in S content. 

Additionally, taking V-rTCNQ and Mn-rTCNQ as examples, the DOS of the adsorp-tion systems were calculated and are plotted in Figure 3b. The metallic features of V-rTCNQ and Mn-rTCNQ are well maintained during the whole process, ensuring fast elec-tron transportation during the redox process of LiPSs. In addition, the tiny contribution of S_8_ to the electronic states near the Fermi level is observed, manifesting the physical adsorption features. As Li ratio increases in the LiPSs, the *s* orbitals of lithium and *p* orbitals of N atoms have significant hybridization, verifying the formation of Li-N chem-ical bonds.

### 2.4. Charging-Discharging Catalytic and Li-ion Diffusion Capability

**Figure 4:**
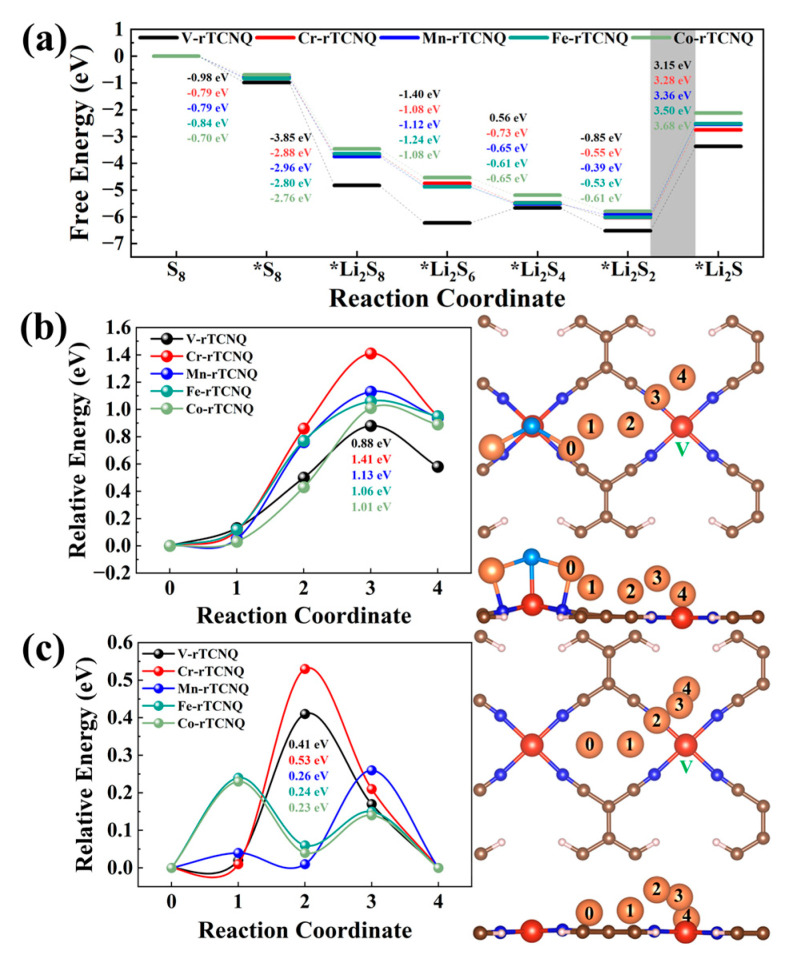
(**a**) The Gibbs free energy ladder diagram of SRRs process of V-rTCNQ, Cr-rTCNQ, Mn-rTCNQ, Fe-rTCNQ and Co-rTCNQ structures. (**b**,**c**) The relative energy profiles of TM-rTCNQ (TM = V, Cr, Mn, Fe and Co) and migration path of Li_2_S decomposition or Li-ion diffusion on V-rTCNQ, respectively.

In addition to the anchoring effect for S_8_/LiPSs, we also systematically investigated the catalytic performance of TM-rTCNQ for the whole charging-discharging process of LiSBs by simulation of the oxidation and reduction reactions of elemental sulfur, as well as the kinetic rate of Li-ion diffusion [2,12,57]. In this regard, the energy barriers of three dynamic processes were calculated based on DFT methods, and the summarized results are shown in Figure 4 and Figure S7, as well as in Tables S2 and S3. The Gibbs free energy ladder diagram of the sulfur reduction reactions (SRRs) process (discharging process) in Figure 4a shows that the reaction process from S_8_ to *Li_2_S_8_ is basically exothermic, except for the V-rTCNQ conversion of *Li_2_S_6_ to *Li_2_S_4_, whereas the conversion process from *Li_2_S_2_ to *Li_2_S is all endothermic. The rate-limiting step (RLS) of the whole catalytic reaction process is the *Li_2_S_2_ → *Li_2_S, due to the maximum Gibbs free energy change (Δ*G*6). As displayed in Figure 4a, Δ*G*6 values for TM-rTCNQ (V, Cr, Mn, Fe and Co) are 3.15 eV, 3.28 eV, 3.36 eV, 3.50 eV and 3.68 eV respectively, and V-rTCNQ exhibits the smallest Δ*G* (3.15 eV) for RLS. 

The first-step reaction of the charging process (Li_2_S dissociation: *Li_2_S → *LiS + Li^+^ + e^−^) and the transition states of the Li-ion diffusion were also calculated using the CI-NEB method. The results show that V-rTCNQ and Co-rTCNQ have the lowest Li_2_S decompo-sition and Li-ion diffusion barriers, respectively. The migration pathways of V-rTCNQ are plotted in the right side of Figure 4b, c (0 is the initial-state position and 4 is the final-state position), and those of other structures are shown in Figure S7. Figure 4b, c shows the minimum energy paths for Li diffusion on the surface of TM-rTCNQ (TM = V, Cr, Mn, Fe and Co). The reaction coordinates 1–3 stand for the intermediate images along the reaction path and reaction coordinate 3 is the transition state for the diffusion of Li-ions. It is evi-dent that the dissociated single Li-ion moves far away from the center TM atoms to the N atoms of adjacent N_4_ centers, accompanied by the fracture of the Li−S bonds. As shown in Table 1, our calculated results predict that un-synthesized V-rTCNQ and synthesized Mn-rTCNQ can be applied as sulfur hosts with excellent charging-discharging performance, and their RLS and Li_2_S dissociation energy barriers are comparable with, or even lower than, those of SACs in the recent literature. The Li-ion diffusion behaviors of TM-rTCNQ (TM = V, Mn, Fe and Co) are also comparable with some other types of 2D material (the Li diffusion barrier is 0.66 eV for β_12_-borophene [58], 0.38 eV for an InP_3_ monolayer [59], 0.33 eV for graphene [60], 0.43 eV for C_3_N [61], 0.35 eV for TiPc [12], etc.).

All in all, considering that V-rTCNQ has the most suitable adsorption strength for S_8_/LiPSs clusters among all TM-rTCNQ, the lowest charging-discharging reaction energy barrier and acceptable Li-ion diffusion, it exhibits very promising value for LiSBs. Although the binding strength of Mn-rTCNQ to polysulfides is not the strongest, the en-ergy barrier of its discharging reaction is significantly lower than that of Fe-rTCNQ and Co-rTCNQ, and charging reaction performance is also batter than that of Cr-rTCNQ and is comparable with that of Fe-rTCNQ and Co-rTCNQ. What’s more, the Li-ion diffusion energy barrier of Mn-rTCNQ is even lower than that of V-rTCNQ and Cr-rTCNQ, and it is very close to that of the best Co-rTCNQ. More importantly, this material has been pre-pared in experiments, thus also shows great application potential.

### 2.5. AIMD Simulation Results

**Figure 5:**
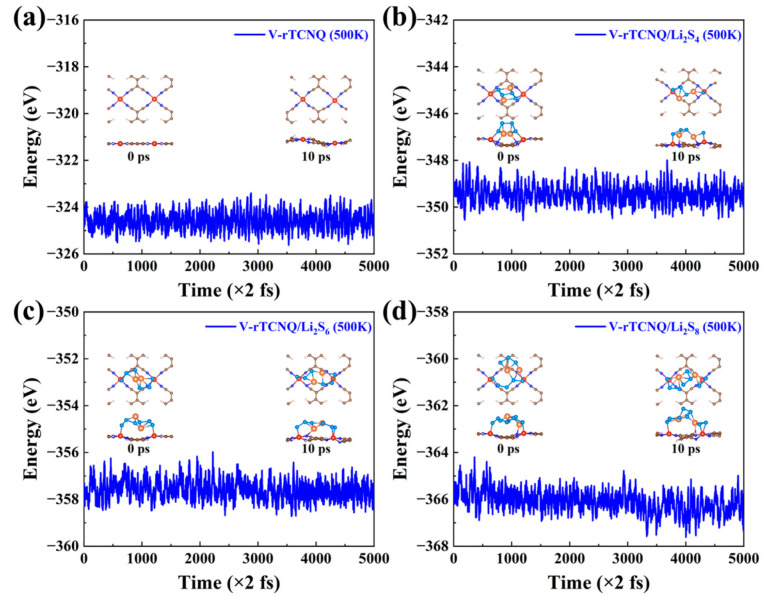
The AIMD simulation results of the V-rTCNQ structure (**a**) and adsorption systems of V-rTCNQ/Li_2_S_4_ (**b**), V-rTCNQ/Li_2_S_6_ (**c**), V-rTCNQ/Li_2_S_8_ (**d**) at 500 K and 10 ps.

In particular, long-term stability is a basic and vital element for application as a host material in LiSBs. Theoretically, AIMD simulations are further performed to ensure the thermal stability of TM-rTCNQ (TM = V, Cr, Mn, Fe and Co) substrates and adsorption systems. As representatives, TM-rTCNQ (TM = V, Cr, Mn, Fe and Co) monolayers and the adsorption systems of V-rTCNQ/Li_2_S_4_ (Li_2_S_6_, Li_2_S_8_) were chosen to implement AIMD sim-ulations with the NVT ensemble at 500 K for 10 ps. As displayed in Figure 5a and Figure S8, the geometries of the TM-rTCNQ (TM = V, Cr, Mn, Fe and Co) monolayers are well preserved when the temperature increases to 500 K. In addition, AIMD simulations were also carried out to check the overall thermal stability of the long-chain LiPSs (Li_2_S_8_, Li_2_S_6_ and Li_2_S_4_)- terminated V-rTCNQ systems. As detailed in Figure 5b–d, the results reveal that the sys-tems with adsorbed long-chain LiPSs and their hosts have no significant bond breakage and structural distortion. Our AIMD results show that the TM-rTCNQ monolayers and the adsorption systems of V-rTCNQ for soluble Li_2_S_4_ (Li_2_S_6_, Li_2_S_8_) possess excellent ther-mal stability. Hence, V-rTCNQ can be further prepared and has great application poten-tial as a substrate that can greatly improve LiSBs performance. Last, but not least, synthe-sized Mn-rTCNQ is also extremely ready to be used in the experimental exploration of LiSBs.

## 3. Computational Details and Methods

### 3.1. Computational Details

In our work, all spin-polarized first-principles calculations were completed by the Vienna ab-initio Simulation Package (VASP) based on the density functional theory (DFT) [63]. The ion-electron interactions were described by the projector augmented wave (PAW) method [64] and the general gradient approximation (GGA) [65,66] in the Perdew–Burke–Ernzerhof (PBE) [67] form was used. The DFT-D3 [68] semi-empirical correction scheme of Gimme was adopted to treat the van der Waals (vdW) interaction between S_8_/LiPSs and substrates. S_8_ and Li_2_S*_x_* (*x* = 1, 2, 4, 6, 8) clusters were placed in a box of 20 × 20 × 20 Å^3^ to fully relax. A supercell, consisting of 2 × 1× 1 two-dimensional (2D) TM-rTCNQ monolayers, was utilized to anchor S_8_/LiPSs, and the vacuum layer was set as 20 Å to eliminate the interactions between two periodic units. The simulation was run with a cutoff energy of 520 eV throughout the computations. These settings ensure convergence of the total energies and force within 10^-5^ eV and 0.02 eV/Å, respectively. The energy bar-riers of Li_2_S decomposition and Li-ion diffusion were calculated using the climbing image nudged elastic band (CI-NEB) method [69,70]. Bader-charge analysis [71] was conducted to simulate the amount of charge transfer between TM-rTCNQ and S_8_/LiPSs. Finally, ab-initio molecular dynamics (AIMD) simulations with a total time of 10 ps (5000 steps and step size set as 2 fs) were conducted to evaluate the thermal stability of TM-rTCNQ sub-strates and the adsorption systems of V-rTCNQ/Li_2_S_4_ (Li_2_S_6_ and Li_2_S_8_).

### 3.2. Computational Methods

Structural stability is one of the important factors for theoretically predicting the fea-sibility of experimental preparation of catalytic materials. It is understood that the TM-rTCNQ formed by the combination of TM and TNCQ can be fully judged through the adsorption energy (*E*_a_), cohesion energy (*E*_c_) and the difference (*E*_diff_) between them [72,73]. Their detailed definitions are as follows:

(1) $$E_{a}{(\mathrm{TM})=E}_{\mathrm{TM}-\mathrm{rTCNQ}}-{\;E}_{\mathrm{support}}-{\;E}_{\sin\mathrm{gle}-\mathrm{TM}}$$

(2) $$E_{\mathrm{coh}}{(\mathrm{TM})=\mu }_{\mathrm{TM}}-{\;E}_{\sin\mathrm{gle}-\mathrm{TM}}$$

(3) $$E_{\mathrm{diff}}\left(\mathrm{TM}\right){=E}_{a}\left(\mathrm{TM}\right)-{\;E}_{\mathrm{coh}}\left(\mathrm{TM}\right)$$

where $E_{\mathrm{TM}-\mathrm{rTCNQ}}$,${\;E}_{\mathrm{support}}$ and $E_{\mathrm{single-TM}}$ are the total energies of TM-rTCNQ, the sup-ports and isolated single transition metal (TM) atoms (TM = Sc, Ti, V, Cr, Mn, Fe, Co, Ni, Cu and Zn), respectively.$\mu_{\mathrm{TM}}$ are the energies of TM in their stable bulk phase. When $E_{\mathrm{diff}}(\mathrm{TM})\;<\;0$, TM-rTCNQ can be considered as an experimental preparation and won’t easily agglomerate into clusters.

In order to accurately evaluate the anchoring ability of TM-rTCNQ structures to S_8_/LiPSs, the formula of adsorption energy (*E*_ads_) is defined as [6,22]:

(4) $$E_{\mathrm{ads}}{=E}_{{\mathrm{TM}-\mathrm{rTCNQ}+S}_{8}/\mathrm{LiPSs}}-{\;E}_{\mathrm{TM}-\mathrm{rTCNQ}}-{\;E}_{S_{8}/\mathrm{LiPSs}}$$

where $E_{{\mathrm{TM}-\mathrm{rTCNQ}+S}_{8}/\mathrm{LiPSs}}$,${\;E}_{\mathrm{TM}-\mathrm{rTCNQ}}$ and${\;E}_{S_{8}/\mathrm{LiPSs}}$ are the total energies of the adsorption systems of TM-rTCNQ substrates with${\;E}_{S_{8}/\mathrm{LiPSs}}$ clusters, TM-rTCNQ monolayers and isolated S_8_/LiPSs molecules, respectively. According to the above definition, more nega-tive $E_{\mathrm{ads}}$ corresponds to stronger adsorption interaction.

The charge density difference (Δ*ρ*) is used to visualize the electron transfer between TM-rTCNQ and S_8_/LiPSs, and its calculation formula is as follows [12,33]:

(5) $$\Delta {\rho =\rho }_{{\mathrm{TM}-\mathrm{rTCNQ}+S}_{8}/\mathrm{LiPSs}}-{\;\rho }_{\mathrm{TM}-\mathrm{rTCNQ}}-{\;\rho }_{S_{8}/\mathrm{LiPSs}}$$

where $\rho_{{\mathrm{TM}-\mathrm{rTCNQ}+S}_{8}/\mathrm{LiPSs}}$, $\rho_{\mathrm{TM}-\mathrm{rTCNQ}}$ and $\rho_{S_{8}/\mathrm{LiPSs}}$ are the charge density of adsorption systems, substrate materials and polysulfides, respectively.

To quantitatively analyze the degree of vdW and chemical interactions to determine the nature of the substrates’ adsorption of polysulfides, the contribution ratio of vdW (*R*_vdW_) interaction is calculated by the following formula [2,33]:

(6) $$R_{\mathrm{vdW}}=\frac{E_{\mathrm{ads}}^{\mathrm{vdW}}-E_{\mathrm{ads}}^{\mathrm{without}-\mathrm{vdW}}}{{\;E}_{\mathrm{ads}}^{\mathrm{vdW}}}\;\times \;100\%$$

where $E_{\mathrm{ads}}^{\mathrm{with-vdW}}$ and $E_{\mathrm{ads}}^{\mathrm{without-vdW}}$ stand for the adsorption energies with or without vdW correction.

The overall discharge reaction or sulfur reduction reactions (SRRs) process (S_8_ + 16Li^+^ + 16e^−^ → 8Li_2_S) of LiSBs can be depicted by following Equations (7)–(12) [5,6,12,62], where the S_8_/LiPSs species with “*” indicate that they were adsorbed on the TM-rTCNQ. The energy of a single Li-ion and an electron (Li^+^ + e^−^) pair was treated as the energy of a crystalline Li atom.

S_8_ + 16Li^+^ → ^*^S_8_ + 16Li^+^ (∆*G*1) (7)

^*^S_8_ + 16Li^+^ + 2e^−^ → ^*^Li_2_S_8_ + 14Li^+^ (Δ*G*2) (8)

^*^Li_2_S_8_ + 14Li^+^ + 2e^−^ → ^*^Li_2_S_6_ + Li_2_S_2_ + 12Li^+^ (Δ*G*3) (9)

^*^Li_2_S_6_ + Li_2_S_2_ + 12Li^+^ + 2e^−^ → ^*^Li_2_S_4_ + 2Li_2_S_2_ + 10Li^+^ (Δ*G*4) (10)

^*^Li_2_S_4_ + 2Li_2_S_2_ + 10Li^+^ + 2e^−^ → ^*^Li_2_S_2_ + 3Li_2_S_2_ + 8Li^+^ (Δ*G*5) (11)

^*^Li_2_S_2_ + 3Li_2_S_2_+ 8Li^+^ + 8e^−^ → ^*^Li_2_S + 7Li_2_S (Δ*G*6) (12)

The Gibbs free energy (ΔG) for above each elementary step was calculated as follows [5,12,14,62]:

(13) $$\Delta G=\Delta E_{\mathrm{DFT}}+\Delta E_{\mathrm{ZPE}}-\;T\Delta S\;-\;\mathrm{neU}$$

Herein, $\Delta E_{\mathrm{DFT}}$ is directly obtained by electronic energy difference of each elemen-tary reaction. $\Delta E_{\mathrm{ZPE}}$ and TΔS stand for zero-point energy and entropy change at 298.15 K, respectively. n and U are the number of electrons transferred and the applied voltage. For the battery systems, the$\;\Delta E_{\mathrm{ZPE}}$ and TΔS can be ignored in the calculation [11,12,62], therefore, when U = 0 V, ΔG ≈ $\Delta E_{\mathrm{DFT}}$. 

## 4. Conclusions

In our work, we systematically evaluated the potential application of all 3*d* family TM-rTCNQ (TM = Sc, Ti, V, Cr, Mn, Fe, Co, Ni, Cu and Zn) configurations in LiSBs. Among them, TM-rTCNQ (TM = V, Cr, Mn, Fe and Co) structures not only have excellent conductivity and structural stability, but also can produce appropriate adsorption strength for S_8_/LiPSs to inhibit shuttle effects. V-rTCNQ is predicted to have excellent charging-discharging catalytic performance because of its lower RLS barrier (3.15 eV) and Li_2_S dissociation barrier (0.88 eV) than many other 2D materials, as well as having a con-siderably low Li-ion diffusion barrier (0.41eV). AIMD simulation results reveal that the TM-rTCNQ (TM = V, Cr, Mn, Fe and Co) substrates and the adsorption system for soluble LiPSs onto V-rTCNQ are highly evaluated for their good thermodynamic stability at high temperatures. Additionally, the synthesized Mn-rTCNQ is also very worthy of further experimental confirmation and exploration. However, we also realize that obtaining ex-cellent substrate materials from a variety of constructed SACs is a relatively complicated process, which also indicates that future theoretical work needs to explore more rapid methods for comprehensively predicting the performance of LiSBs, such as high-through-put screening or machine learning. Nevertheless, our theoretical research still proposes a new candidate for exploring novel MOF catalysts with outstanding LiSBs catalytic perfor-mance and has great guiding significance for further experimental work.

## Figures and Tables

**Table 1:** The RLS and Li_2_S dissociation barrier of V/Mn-rCTNQ and other SACs in some recent literatures.

Structures	RLS/eV	Energy Barrier (Li_2_S Dissociation) /eV
V-rTCNQ	3.15	0.88
Mn-rTCNQ	3.36	1.13
VN_4_@graphene [5]	4.24	0.70
TiPc [12]	4.33	0.86
VPc [12]	4.43	1.04
Ti-SnSe [62]	5.47	0.91

## Data Availability

Not applicable.

## Supplementary Materials

The following supporting information can be downloaded at: https://www.mdpi.com/article/10.3390/molecules28052389/s1, Figure S1: The fully optimized unit cell of TM-rTCNQ (TM = Sc, Ti, V, Cr, Mn, Fe, Co, Ni, Cu and Zn) monolayers; Figure S2: The DOS diagrams of TM-rTCNQ (TM = Sc, Ti, Cr, Mn, Fe, Co, Ni, Cu and Zn) unit cell, including the total DOS (TDOS), the projected DOS of *d* orbital of TM(TM_d), *p* orbital of C and N (C_p and N_p), as well as *s* orbital of H (H_s). Fermi-level is at the position of gray dotted line; Figure S3: The stable adsorption configurations and energies of 1,3-dioxolane (DOL) and 1,2-dimethoxyethane (DME) solvent molecules for soluble polysulfides (Li_2_S_4_, Li_2_S_6_, and Li_2_S_8_); Figure S4: The most stable adsorption configurations and energies of TM-rTCNQ (TM = Sc, Ti, V, Cr, Mn, Fe, Co, Ni and Cu) for S_8_/LiPSs clusters in the Z1 adsorption pattern; Figure S5: The most stable adsorption configurations and energies of TM-rTCNQ (TM = Sc, Ti, V, Cr, Mn, Fe, Co, Ni and Cu) for S_8_/LiPSs clusters in the Z2 adsorption pattern; Figure S6: The charge density differences and the charge transfer amount of the adsorption systems of TM-rTCNQ (TM = Cr, Mn, Fe and Co) for S_8_/LiPSs; Figure S7: The Li_2_S decomposition (a) and Li^+^ diffusion (b) pathways of TM-rTCNQ (TM = Cr, Mn, Fe and Co) structures; Figure S8: The AIMD simulation results of TM-rTCNQ (TM = Cr, Mn, Fe and Co) substrates at 500 K and 10 ps; Table S1: The lattice constants and the magnetic moments (Mag) of the unit cell of TM-rTCNQ (TM = Sc, Ti, V, Cr, Mn, Fe, Co, Ni, Cu and Zn) monolayers; Table S2: The relative energies of Li_2_S dissociation pathways on TM-rTCNQ (TM = V, Cr, Mn, Fe and Co) monolayers; Table S3: The relative energies of Li^+^ diffusion pathways on TM-rTCNQ (TM = V, Cr, Mn, Fe and Co) monolayers.
