# Supplementary material for: Rectangular Transition Metal-rTCNQ Organic Frameworks Enabling Polysulfide Anchoring and Fast Electrocatalytic Activity in Li-Sulfur Batteries: A Density Functional Theory Perspective
Source: Molecules. 2023 Mar 5;28(5):2389. doi: 10.3390/molecules28052389 (PMC10005228; doi:10.3390/molecules28052389)
Supplement: Supplementary file 1 [file molecules-28-02389-s001.zip › molecules-2210920-supplementary.pdf]

molecules

MDPI

Supplementary Materials

Rectangular Transition Metal-rTCNQ Organic Frameworks Enabling Polysulfide Anchoring and Fast Electrocatalytic Activity in Li-sulfur Batteries: A Density Functional Theory Perspective

Jie-Zhen Xia [1,2,3,†], Lu-Chao Zhao [1,2,3,†], Man-Hua Liao [1,2,3,†] and Qi Wu [1,2,3,*]

1 School of Science, Tibet University, Lhasa 850000, China; xia_zangda@163.com (J.-Z.X.); zlc_zangda@163.com (L.-C.Z.); liaozangda@163.com (M.-H.L.)
2 Institute of Oxygen Supply, Center of Tibetan Studies (Everest Research Institute), Tibet University, Lhasa, 850000, China
3 Key Laboratory of Cosmic Rays, Tibet University, Ministry of Education, Lhasa 850000, China
* Correspondence: wuqi@utibet.edu.cn
† The authors contributed equally to this work.

The supplementary materials include two parts (**1. Supplementary figures and 2. Supplementary tables**).

1. Supplementary Figures

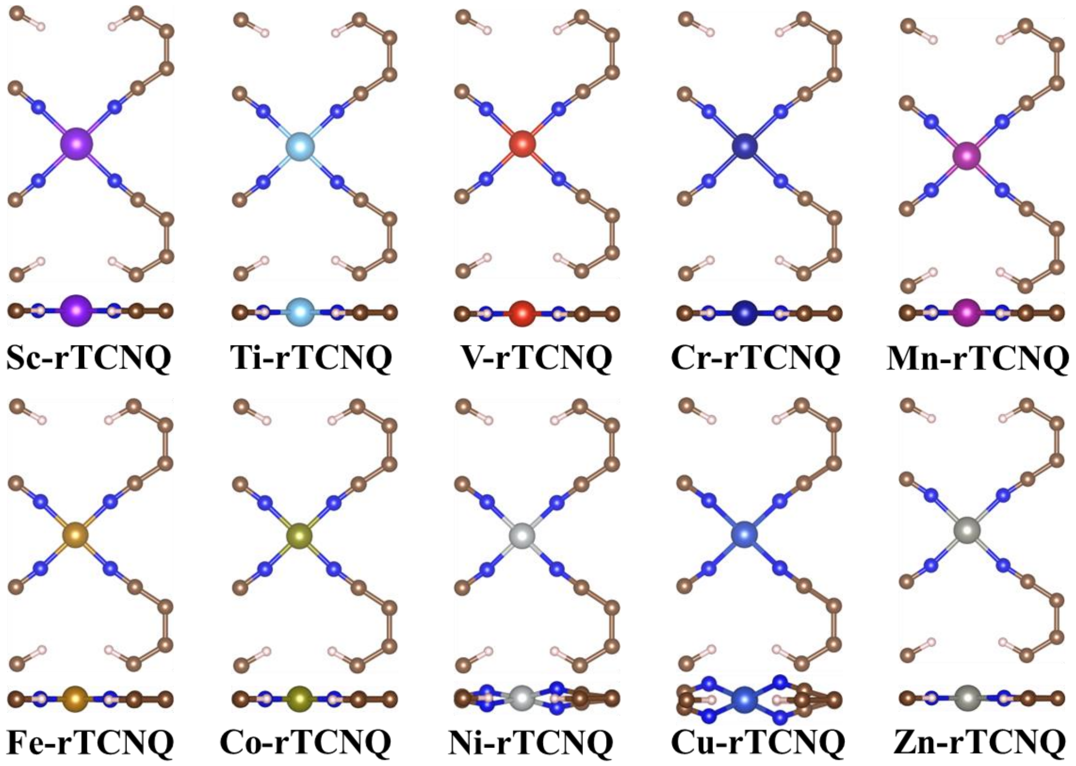

Figure S1. The fully optimized unit cell of TM-rTCNQ (TM = Sc, Ti, V, Cr, Mn, Fe, Co, Ni, Cu and Zn) monolayers.

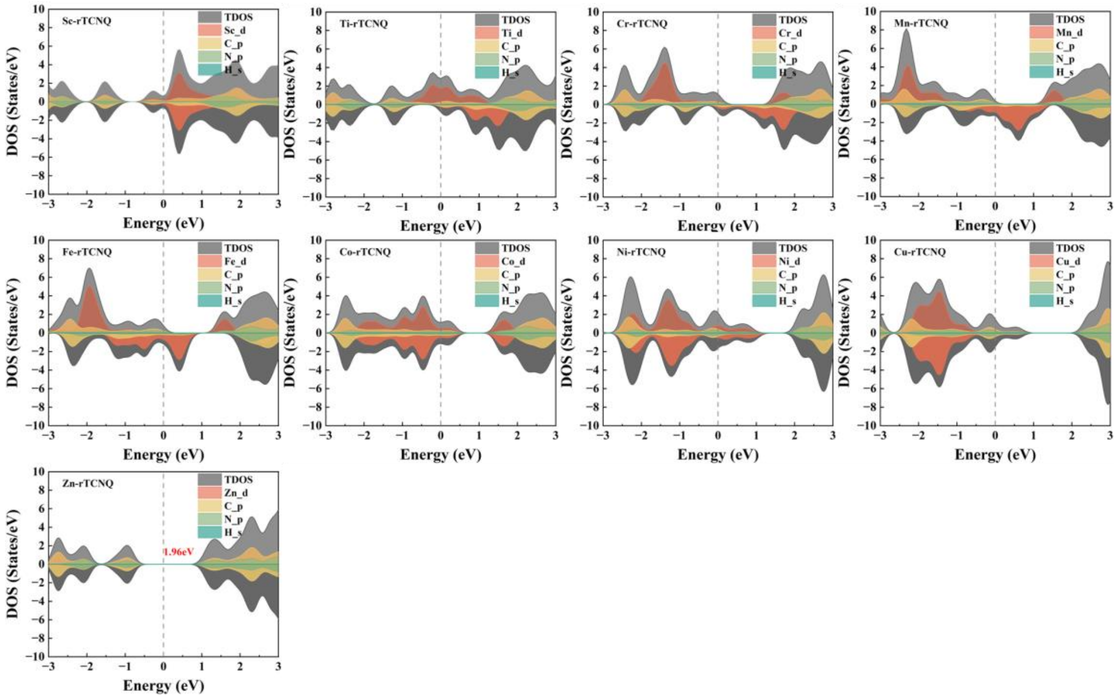

Figure S2. The DOS diagrams of TM-rTCNQ (TM = Sc, Ti, Cr, Mn, Fe, Co, Ni, Cu and Zn) unit cell, including the total DOS (TDOS), the projected DOS of *d* orbital of TM(TM_d), *p* orbital of C and N (C_p and N_p), as well as *s* orbital of H (H_s). Fermi-level is at the position of gray dotted line.

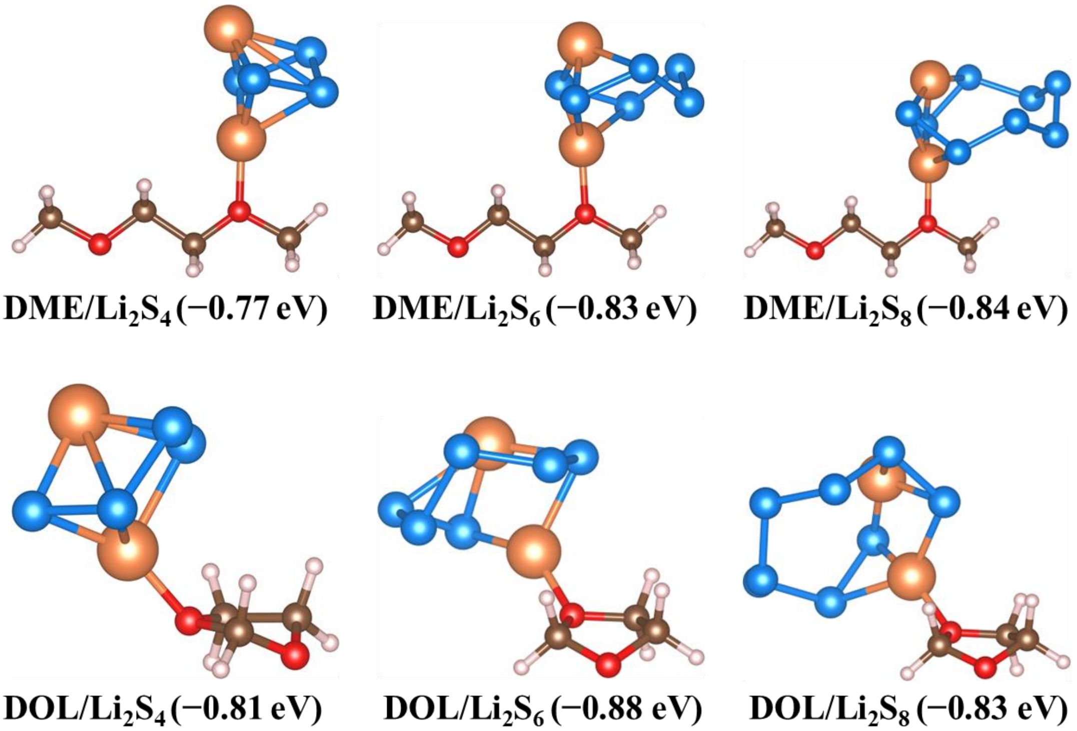

Figure S3. The stable adsorption configurations and energies of 1,3-dioxolane (DOL) and 1,2-dimethoxyethane (DME) solvent molecules for soluble polysulfides (Li_2S_4, Li_2S_6, and Li_2S_8).

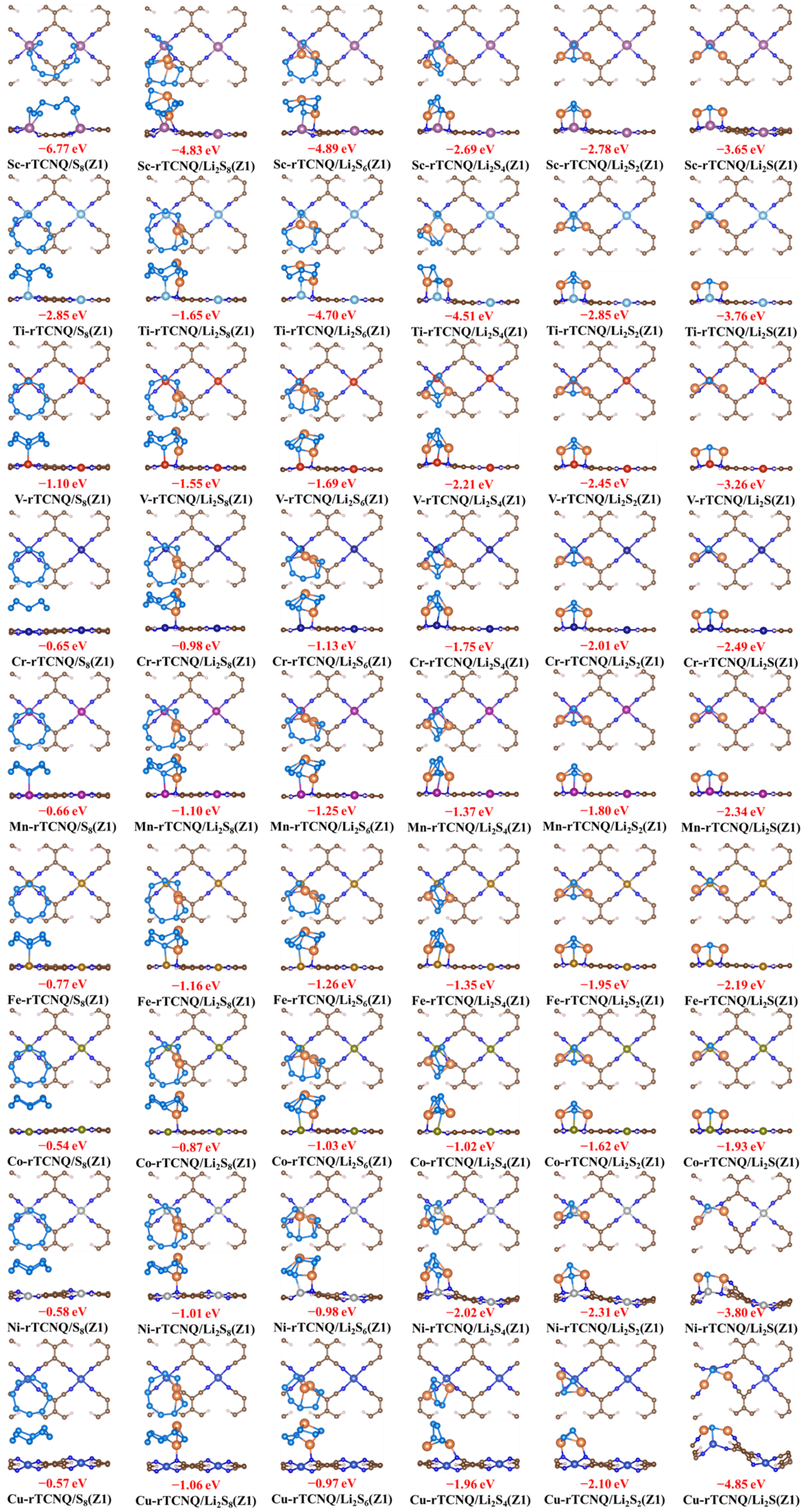

Figure S4. The most stable adsorption configurations and energies of TM-rTCNQ (TM = Sc, Ti, V, Cr, Mn, Fe, Co, Ni and Cu) for S_8/LiPSs clusters in the Z1 adsorption pattern.

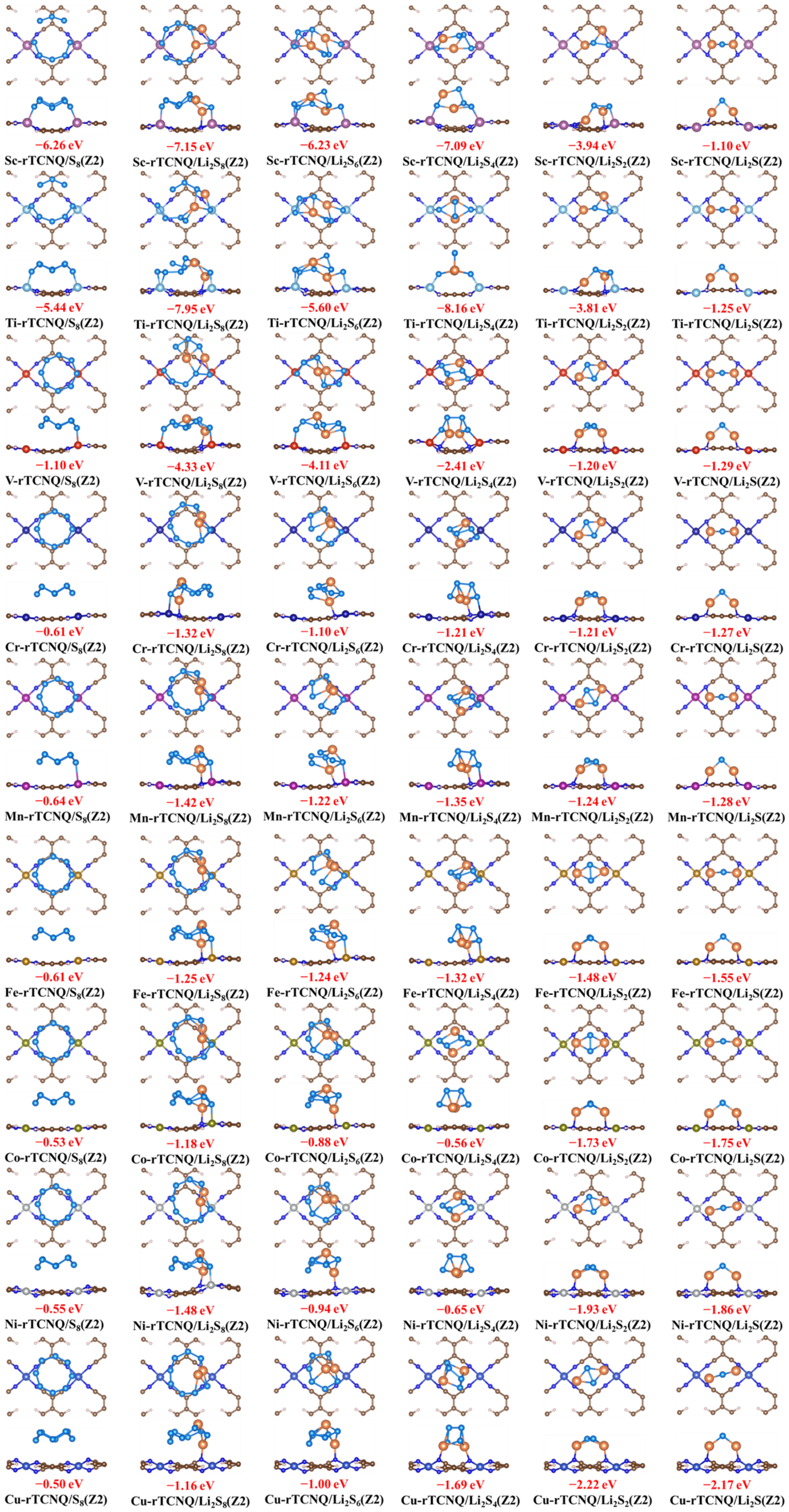

Figure S5. The most stable adsorption configurations and energies of TM-rTCNQ (TM = Sc, Ti, V, Cr, Mn, Fe, Co, Ni and Cu) for S_8/LiPSs clusters in the Z2 adsorption pattern.

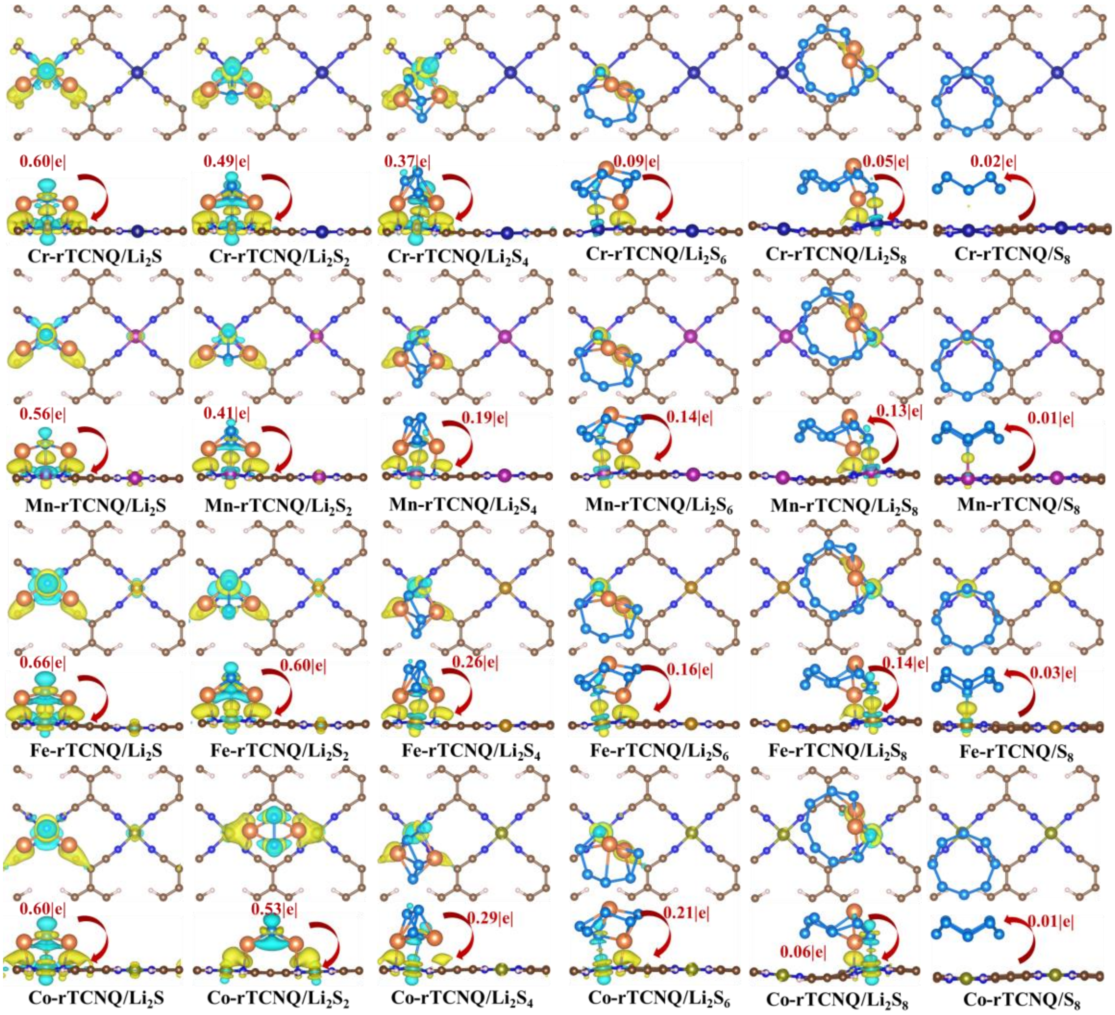

Figure S6. The charge density differences and the charge transfer amount of the adsorption systems of TM-rTCNQ (TM = Cr, Mn, Fe and Co) for S_8/LiPSs.

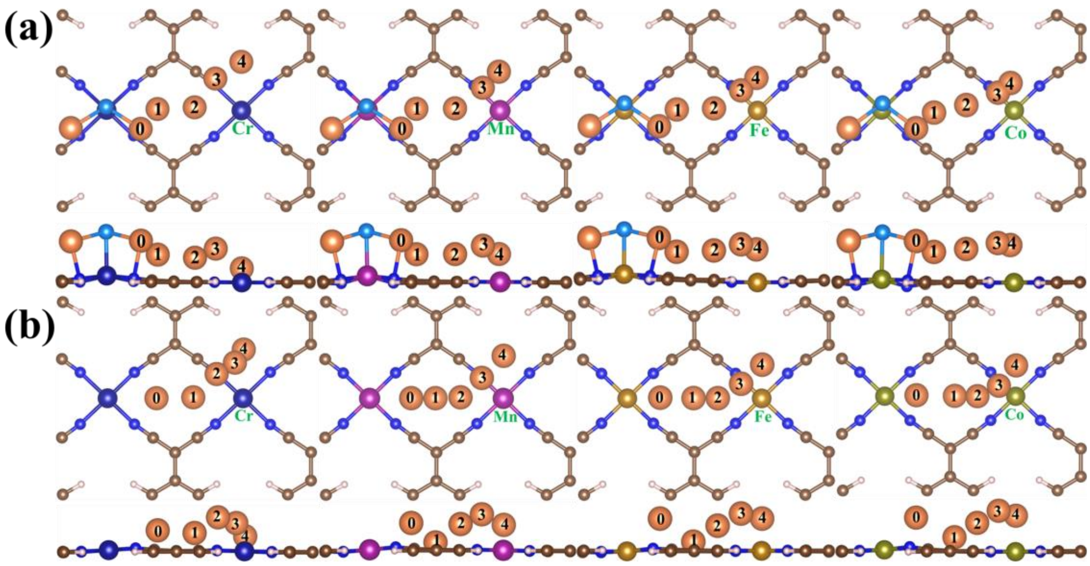

Figure S7. The Li_2S decomposition (a) and Li^+ diffusion (b) pathways of TM-rTCNQ (TM = Cr, Mn, Fe and Co) structures.

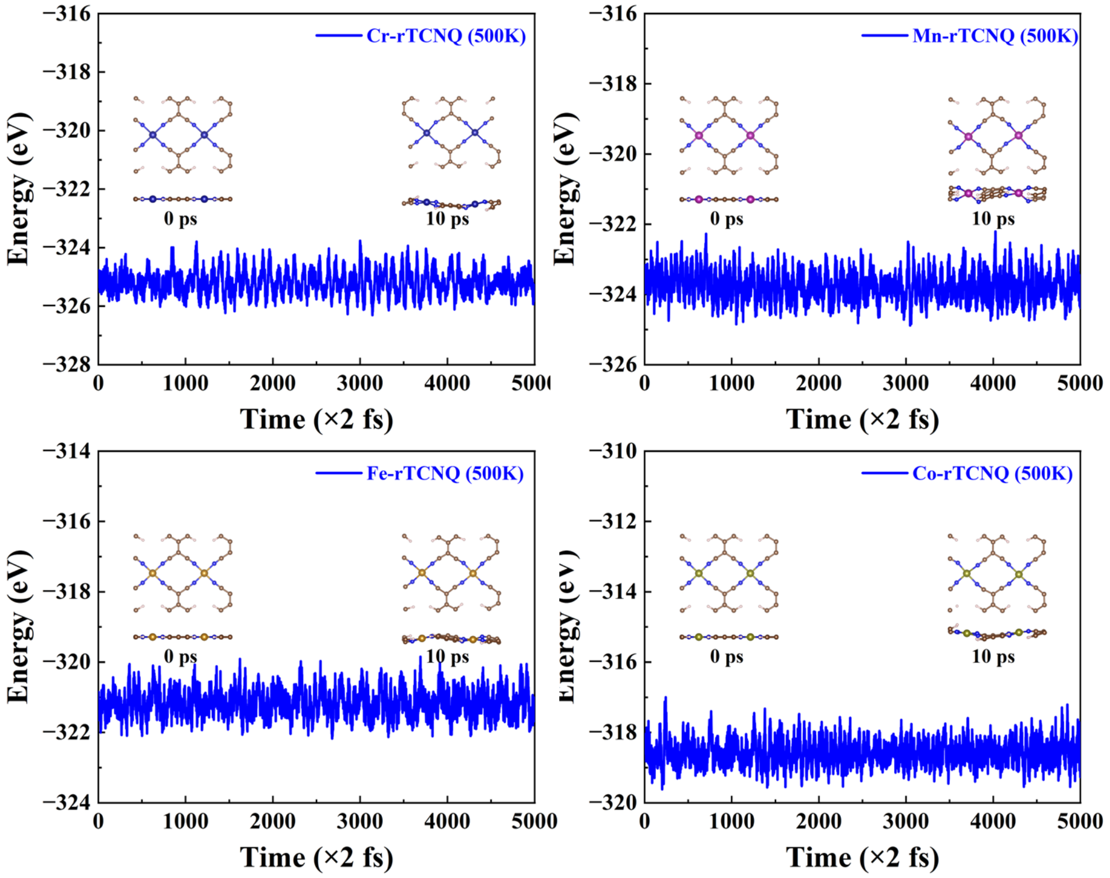

Figure S8. The AIMD simulation results of TM-rTCNQ (TM = Cr, Mn, Fe and Co) substrates at 500 K and 10 ps.

2. Supplementary Tables

Table S1. The lattice constants and the magnetic moments (Mag) of the unit cell of TM-rTCNQ (TM = Sc, Ti, V, Cr, Mn, Fe, Co, Ni, Cu and Zn) monolayers.

TM-rTCNQ	Lattice Constant: a (Å)	Lattice Constant: b (Å)	Mag(μb)
Sc-rTCNQ	7.165	11.783	0.000
Ti-rTCNQ	7.155	11.568	1.873
V-rTCNQ	7.115	11.434	2.993
Cr-rTCNQ	7.062	11.418	3.888
Mn-rTCNQ	6.913	11.361	2.988
Fe-rTCNQ	6.884	11.260	1.899
Co-rTCNQ	6.809	11.197	0.000
Ni-rTCNQ	6.827	11.196	0.129
Cu-rTCNQ	6.784	11.119	0.000
Zn-rTCNQ	7.184	11.393	0.000

Table S2. The relative energies of Li_2S dissociation pathways on TM-rTCNQ (TM = V, Cr, Mn, Fe and Co) monolayers.

Structures	0/eV	1/eV	2/eV	3/eV	4/eV
V-rTCNQ	0	0.13	0.50	0.88	0.58
Cr-rTCNQ	0	0.11	0.86	1.41	0.95
Mn-rTCNQ	0	0.05	0.76	1.13	0.94
Fe-rTCNQ	0	0.12	0.77	1.06	0.95
Zn-rTCNQ	0	0.03	0.43	1.01	0.89

Table S3. The relative energies of Li^+ diffusion pathways on TM-rTCNQ (TM = V, Cr, Mn, Fe and Co) monolayers.

**Structures**	**0/eV**	**1/eV**	**2/eV**	**3/eV**	**4/eV**
V-rTCNQ	0	0.02	0.41	0.17	0
Cr-rTCNQ	0	0.01	0.53	0.21	0
Mn-rTCNQ	0	0.04	0.01	0.26	0
Fe-rTCNQ	0	0.24	0.06	0.15	0
Zn-rTCNQ	0	0.23	0.04	0.14	0
